# Identifying patterns of lifestyle behaviours among children of 3 years old

**DOI:** 10.1093/eurpub/ckaa109

**Published:** 2020-07-12

**Authors:** Lu Wang, Wilma Jansen, Amy van Grieken, Eline Vlasblom, Magda M Boere-Boonekamp, Monique P L’Hoir, Hein Raat

**Affiliations:** c1 Department of Public Health, Erasmus University Medical Center, Rotterdam, The Netherlands; c2 Department of Social Development, City of Rotterdam, Rotterdam, The Netherlands; c3 TNO Child Health, Leiden, The Netherlands; c4 Department of Health Technology and Services Research, Technical Medical Center, University of Twente, Enschede, The Netherlands; c5 Department of Agrotechnology and Food Sciences, Subdivision Human Nutrition, Wageningen University & Research, Wageningen, The Netherlands

## Abstract

**Background:**

To identify the patterns of lifestyle behaviours in children aged 3 years, to investigate the parental and child characteristics associated with the lifestyle patterns, and to examine whether the identified lifestyle patterns are associated with child BMI and weight status.

**Methods:**

Cross-sectional data of 2090 children 3 years old participating in the Dutch BeeBOFT study were used. Child dietary intakes, screen times and physical activity were assessed by parental questionnaire, and child weight and height were measured by trained professionals according to a standardized protocol. Latent class analysis was applied to identify patterns of lifestyle behaviours among children.

**Results:**

Three subgroups of children with distinct patterns of lifestyle behaviours were identified: the ‘unhealthy lifestyle’ pattern (36%), the ‘low snacking and low screen time’ pattern (48%) and the ‘active, high fruit and vegetable, high snacking and high screen time’ pattern (16%). Children with low maternal educational level, those raised with permissive parenting style (compared those with authoritative parents), and boys were more likely be allocated to the ‘unhealthy lifestyle’ pattern and the ‘active, high fruit and vegetable, high snacking and high screen time’ pattern (*P* < 0.05). No association was found between the identified lifestyle patterns and child BMI z-score at age 3 years.

**Conclusions:**

Three different lifestyle patterns were observed among children aged 3 years. Low maternal educational level, permissive parenting style and male gender of the child were associated with having unhealthy lifestyle patterns for the child.

## Introduction

Childhood obesity is one of the major public health concerns nowadays due to its high prevalence and adverse physical and psychological outcomes.^1–3^ Children’s lifestyle behaviours, including high intake of energy-dense nutrition-low foods (e.g. high intake of sugar-sweetened beverages, unhealthy snacks), high levels of sedentary behaviours (e.g. television viewing, computer use) and low level of physical activity are known to contribute to energy imbalance and therefore increase the risk of child overweight and obesity.^4–6^

Increasing evidence suggests that certain overweight-related lifestyle behaviours may co-occur or cluster in some subgroups of children.^7–11^ For example, Leech et al.^8^ identified three subgroups of children with distinct lifestyle patterns among Australian children 5–6 years (*n* = 362), the ‘most healthy’ group, the ‘energy-dense consumers who watch TV’ group and the ‘high sedentary behaviour/low moderate-to-vigorous PA’ group. Miguel-Berges et al.^9^ identified six subgroups of children with different lifestyle behaviour patterns among children aged 4–6 years from six European countries (*n* = 5357), one of which was characterized by the co-existence of unhealthy dietary intakes, high screen time and low physical activity level. Understanding the co-occurrence patterns of lifestyle behaviours is important, as multiple overweight-related lifestyle behaviours may have a synergic effect on the development of overweight and obesity.^7^^,^^12^ In addition, such information can inform intervention developers about which behavioural factors need to be targeted simultaneously. However, few studies have examined the co-occurring or clustering patterns of lifestyle behaviours among children younger than 5 years.^9^

Parents play a pivotal role in shaping children’s lifestyle behaviours,^13^ especially for younger children. Insight in parental- and child-related factors associated with children’s lifestyle patterns will contribute to the development of effective interventions. Parenting style is one factor that may contribute to the development of children’s lifestyle behaviours.^13^^,^^14^ However, studies evaluating parenting style as a determinant of children’s lifestyle patterns are lacking. To address these gaps, this study aimed to (i) examine the clustering patterns of lifestyle behaviours among preschool children aged 3 years in a population-based sample from the Netherlands, (ii) examine the association of parental- and child-related factors with lifestyle patterns, including sociodemographic characteristics and parenting style and (iii) examine the association of the identified lifestyle patterns with children’s BMI and weight status.

## Methods

### Study population

Data from the BeeBOFT study were used. The BeeBOFT study is a cluster randomized controlled trial for the primary prevention of overweight among children.^15^^,^^16^ A total of 51 regional Youth Health Care (YHC) teams covering both rural and urban regions in the Netherlands participated in the study and were randomly allocated to the three study arms. The Medical Ethics Committee of the Erasmus Medical Center concluded that the Dutch Medical Research Involving Human Subjects Act (in Dutch: Wet medisch-wetenschappelijk onderzoek met mensen) did not apply to the research proposal of BeeBOFT (proposal number MEC-2008-250), and therefore had no objection to the execution of this study.

From January 2009 through September 2010, 7985 parent–child dyads were invited to participate in the study by the 51 regional YHC teams during the first home-visit at 2–4 weeks after child birth. A total of 3003 parent–child dyads provided written informed consent for participation in this 3-year study and returned the baseline questionnaire. The parents were invited to fill in a questionnaire at child age 6, 14 and 36 months, respectively. The questionnaires were instructed to be filled by the main caregivers (mother, father or others) of the child. For this study, we used data on children’s lifestyle behaviours at age 36 months. In total, parents of 2253 children returned the questionnaire at child age 36 months, among which 94% were filled by mothers. For this study, children with no missing value on the lifestyle variables (*n* = 2090) were included for analyses.

Non-response analysis shows that compared with children included in this study, children excluded from this study due to non-response for the parental questionnaire and missing values were more likely to have lower maternal educational level, and non-Dutch ethnic background (*P *<* *0.01, see Supplementary table S1 for detailed data).

## Measurements

### Children’s lifestyle behaviours

Children’s lifestyle behaviours including the consumption of sugar-containing beverages, unhealthy snack foods, fruits, vegetables, physical activity and screen time were measured by parental questionnaires at child age 36 months, using Dutch questionnaires that have been used in previous studies (see Supplementary table S2 for detail).^17^^,^^18^ When answering the questions, parents were asked to report the average condition during the past 4 weeks. Each lifestyle variable was dichotomized into favourable vs. unfavourable categories according to current nutrition and physical activity guidelines.^19–24^ Children who were reported to consume sugar-containing drinks for >2 cups per day were classified as having a high consumption of sugar-containing drinks.^23^ Children who were reported to eat unhealthy snacks for two servings or more per day were classified as having a high consumption of unhealthy snacks.^23^ Children who were reported to have fresh fruit for one serving or less were classified as having a low fruit consumption.^23^ Children who were reported to have vegetable for one serving spoon or less were classified as having a low vegetable consumption.^23^ Children who were active for 5 or more days per week and >1 h per day were classified as physically active, while those who did not meet this criterion were classified as having a low level of physical activity.^19^ Children who were reported to have >1 h of screen time on average per day were classified as having excessive screen time.^20^^,^^21^

### Sociodemographic characteristics

Information on maternal age and educational level, parity and child’s gender and ethnic background were assessed by baseline questionnaire. We used maternal body weight and height reported at child age 36 months (or at child age 6 months in case of missing) to calculate maternal BMI (weight in kilograms divided by squared body length in metres). The maternal weight status was then classified as ‘normal’ (BMI < 25), ‘overweight’ (BMI ≥ 25) or ‘obese’ (BMI ≥ 30).

### Parental characteristics

Parenting style was measured at child age 36 months, using a questionnaire used by a previous study.^25^ Two dimensions of parenting were measured: ‘parental warmth’ (6 items), which addresses the frequency with which parents displayed warm affectionate, and ‘parental control’ (5 items), which addresses the frequency with which parents set and enforce clear expectations and limits for their children’s behaviour (see Supplementary table S3 for details). The Cronbach’s alpha for was 0.83 for ‘parental warmth’, and 0.62 for ‘parental control’ dimension. Warmth and control scores were dichotomized at median value in the population and combined to define the four categorical parenting styles.^25^ The combination of high warmth and high control was classified as authoritative; low warmth and high control as authoritarian; high warmth and low control as permissive; and low warmth and low control as neglectful.^25^

The questionnaire at child age 36 months assessed the numbers of half days and full days per week the child using childcares (e.g. daycare centre, childminders, baby sitters, playgroups), which was then categorized as < 1, 1–2.5, 3–3.5, and 4 days and above.

### Child anthropometrics

Child’s weight and height at age 3 years were measured by YHC professionals according to standardized protocols.^26^ Age and gender adjusted BMI z-scores were calculated using the WHO Growth Standard.^27^ At the age of 3 years, each child was classified as being ‘normal weight’, or ‘overweight/obese’ using international Obesity Task Force age- and gender-specific cut-off values.^28^^,^^29^

### Statistical methods

Latent class analysis was applied to identify patterns of lifestyle behaviours, based on dichotomized lifestyle behaviour indicators.^30^^,^^31^ Children that were assigned to the same latent class shared similar patterns of lifestyle behaviours. To identify the optimal number of latent classes, a series of latent class models with a total number of 1–6 classes were evaluated.^30^  Supplementary table S4 presents the model fit statistics of the 1- to 6-class latent class models, including Loglikelihood values, Akaike’s Information Criterion (AIC) and Bayesian Information Criterion (BIC). Both AIC and BIC reached the lowest value at the 3-class model. With further consideration of the model interpretability, eventually the 3-class latent class model was adopted for subsequent analysis.

Multinomial logistic regression models were applied to examine the associations of the parental and child characteristics with the lifestyle patterns identified by the latent class model, including maternal educational level, maternal weight status, parenting styles, child ethnic background, child gender, days using childcare per week and study condition (the intervention or control conditions), adjusted for child exact age (in months), respondent of the questionnaire (mother, father or others) at the time of questionnaire measurement.

The associations of the lifestyle patterns with child BMI z-score and weight status were examined by a linear regression model and a logistic regression model respectively. Both models were adjusted for maternal educational level, maternal weight status, parenting styles, child ethnic background, child gender, child exact age, days using childcare per week, respondent of the questionnaire and study condition.

All the analyses were performed using SAS version 9.4. Latent class analysis was performed with the package ‘PROC LCA’.^30^

## Results

Table 1 presents the characteristics of our study population. Of the 2090 children included in the study, 51% were boys, 84% were of Dutch ethnic background. For the mothers, 33% were categorized as being overweight or obese, and 10% had a low educational level.

**Table 1:** Characteristics of the parents and children in our study population (*n* = 2090)

Variables	*n*	Frequency (%)/mean (SD)
Maternal educational level	2070	
≤4 years secondary school		208 (10.1)
>4 years secondary school or middle level vocational training		732 (35.4)
University or above		1130 (54.6)
Maternal weight status	2079	
Normal		1379 (66.3)
Overweight		519 (25.0)
Obesity		181 (8.7)
Child ethnic background, Dutch	2090	1756 (84.1)
Child gender, male	2057	1059 (51.5)
Child age 36 months, mean (SD)	2090	36.7 (2.2)
Parenting style	2053	
Authoritative		828 (40.3)
Permissive		301 (14.7)
Authoritarian		529 (25.8)
Neglectful		395 (19.2)
Number of days using childcare	2073	
0–0.5 day		160 (7.7)
1–2 days		1056 (50.5)
2.5–3.5 days		582 (27.9)
4 and more days		275 (13.2)
Study condition	2090	
Control condition		730 (34.9)
‘BBOFT+’ Intervention		621 (29.7)
‘E-health4Uth’ Intervention		739 (35.4)

Note: A child’s ethnic background was defined according to the ethnic backgrounds of his/her parents. A parent was classified as non-Dutch if one of his/her own parents was born outside the Netherlands. If one or both of the child’s parents were classified as non-Dutch, that child’s ethnic background was non-Dutch.

Three subgroups of children with distinct lifestyle patterns were identified, which included 36%, 48%, and 16% of the children, respectively (table 2). Children allocated to the ‘unhealthy lifestyle’ pattern (class 1) were more likely to have excessive consumptions of sugar-containing drinks (> 2 cups per day) and unhealthy snacks (>1 serving per day) and insufficient consumptions fruits (<2 serving per day) and vegetables (< 2 serving per day) and were more likely to have excessive screen time (≥1 h per day) and insufficient physical activity level (<1 h per day). Children in the ‘low snacking and low screen time’ pattern (class 2) had low probabilities of having excessive sugar-containing drink and unhealthy snack consumptions, and were less likely to have excessive screen time. Children in the ‘active, high fruit and vegetable, high snacking and high screen time’ pattern (class 3) were more likely to have excessive consumptions of sugar-containing drink and unhealthy snacks and excessive screen time, and less like to report insufficient consumptions of fruit and vegetables and insufficient physical activity level.

**Table 2:** The probabilities of reporting each unfavourable lifestyle behaviours in the total sample and in each lifestyle patterns

Unfavourable lifestyle behaviours	Total sample (*n* = 2090)	Class 1 ‘unhealthy lifestyle’ (*n* = 762, 36%)	Class 2 ‘low snacking and low screen time’ (*n* = 996, 48%)	Class 3 ‘active, high fruit and vegetable, high snacking and high screen time’ (*n* = 332, 16%)	***P-*values** ^a^
Sugar-containing drink consumption >2 cups per day	0.40	0.76	0.02	0.72	<0.001
Unhealthy snack consumption >1 serving per day	0.39	0.65	0.14	0.59	<0.001
Fruit consumption <2 serving per day	0.59	0.80	0.55	0.21	<0.001
Vegetable consumption <2 serving spoons per day	0.68	0.87	0.66	0.32	<0.001
Screen time ≥1 h per day	0.43	0.59	0.25	0.63	<0.001
Physical activity <1 h per day	0.63	0.83	0.64	0.17	<0.001

a The difference between groups was compared using Chi-square test.

With the ‘low snacking and low screen time’ pattern as the reference group, factors significantly (*P *<* *0.05) associated with both the ‘unhealthy lifestyle’ pattern and the ‘active, high fruit and vegetable, high snacking and high screen time’ pattern in the univariate models (table 3) included lower maternal educational level, maternal obesity, permissive parenting style (compare to authoritative parenting style), and male gender of the child. In addition, an authoritarian parenting style was associated with lower probability of being allocated to the ‘active, high fruit and vegetable, high snacking and high screen time’ pattern. In the multivariate model, all the above associations remained significant expected for maternal obesity.

**Table 3:** The associations of parental and child characteristics with children’s lifestyle patterns: results from multinomial logistic regression models (*n* = 2090)

	**Univariate models** ^a^			**Multivariate model** ^b^	
	Class 2, ‘low snacking and low screen time’	Class 1, ‘unhealthy lifestyles’	Class 3, ‘active, high fruit and vegetable, high snacking and high screen time’	Class 1, ‘unhealthy lifestyles’	Class 3, ‘active, high fruit and vegetable, high snacking and high screen time’
		OR (95% CI)	OR (95% CI)	OR (95% CI)	OR (95% CI)
Maternal educational level					
Low vs. high	Ref	1.54 (1.26–1.89)	1.62 (1.23–2.14)	1.51 (1.22–1.88)	1.59 (1.19–2.12)
Middle vs. high	Ref	2.07 (1.47–2.92)**	3.25 (2.18–4.84)***	1.98 (1.37–2.84)**	2.94 (1.91–4.53)***
Maternal weight status					
Overweight vs. normal	Ref	1.28 (1.03–1.60)	1.06 (0.79–1.44)	1.23 (0.97–1.55)	1.00 (0.73–1.37)
Obese vs. normal	Ref	1.49 (1.05–2.10)	1.63 (1.06–2.51)	1.35 (0.94–1.94)	1.46 (0.93–2.29)
Parenting style					
Permissive vs. authoritative	Ref	1.62 (1.20–2.18)**	1.71 (1.19–2.45)***	1.59 (1.17–2.16)**	1.60 (1.10–2.32)**
Authoritarian vs. authoritative	Ref	1.04 (0.82–1.32)	0.64 (0.46–0.90)**	1.03 (0.80–1.31)	0.65 (0.46–0.91)
Neglectful vs. authoritative	Ref	1.18 (0.91–1.54)	0.94 (0.66–1.33)	1.18 (0.90–1.55)	0.89 (0.62–1.28)
Child ethnic background					
Non-Dutch vs. Dutch	Ref	0.77 (0.59–1.00)	0.95 (0.68–1.33)	0.76 (0.58–1.01)	0.88 (0.62–1.27)
Child gender					
Boy vs. girl	Ref	1.26 (1.04–1.52)*	1.30 (1.01–1.67)*	1.25 (1.02–1.52)*	1.28 (0.99–1.66)
Number of days using childcare					
1–2 vs. ≤0.5	Ref	0.97 (0.67–1.41)	0.74 (0.47–1.17)	1.06 (0.71–1.57)	1.01 (0.61–1.68)
2.5–3.5 vs. ≤0.5	Ref	0.78 (0.53–1.15)	0.69 (0.42–1.11)	0.94 (0.62–1.43)	1.07 (0.63–1.84)
≥4 vs. ≤0.5	Ref	1.28 (0.83–1.97)	0.92 (0.53–1.58)	1.40 (0.89–2.21)	1.17 (0.65–2.12)
Study conditions					
‘BBOFT+’ vs. control	Ref	1.11 (0.87–1.40)	0.88 (0.65–1.20)	1.03 (0.80–1.32)	0.86 (0.62–1.20)
‘E-health4Uth’ vs. control	Ref	0.99 (0.79–1.24)	0.86 (0.64–1.15)	1.01 (0.80–1.28)	0.91 (0.66–1.23)

Notes: For both the univariate and the multivariate models, the ‘low snacking, low screen time’ pattern (class 2) was taken as the reference group. All the models were adjusted for child exact age at the time of questionnaire measurement. For maternal educational level, ‘low’ refers to ≤4 years secondary school, ‘middle’ refers to ‘>4 years secondary school or middle level vocational training’, and ‘high’ refers to ‘University or above’.

a For the univariate models, each independent variable (the parental and child characteristics) were entered into the model separately to assess its association with the outcome variable (lifestyle behaviour patterns).

b For the multivariate model, all the independent variables were entered into the model simultaneously to assess the independent association between each parental- and child-related factors and the lifestyle patterns.

*** *P* < 0.0001,

** *P* < 0.001,

* *P* < 0.05.

As shown in table 4, there was no significant association between the lifestyle patterns and child BMI and weight status (overweight vs. normal weight) at age 36 months (all *P *>* *0.1).

**Table 4:** The association between the lifestyle patterns and child BMI z-score and weight status at child age 36 months (*n* = 1309)

	Child BMI z-score		Child overweight	
	Mean (SD)	*ß* (95% CI)^a^	*n* (%)	OR (95% CI)^b^
Class 1,’Unhealthy lifestyle’	−0.45 (0.97)	−0.10 (−0.22, 0.02)	29 (5.92)	0.87 (0.52, 1.46)
Class 2, ‘Low snacking and low screen time’	−0.34 (1.05)	Ref	46 (7.32)	Ref
Class 3, ‘Active, high fruit and vegetable, high snacking, high screen time’	−0.34 (1.02)	0.02 (−0.14, 0.18)	13 (6.81)	0.93 (0.46, 1.89)

Note: All the models adjusted for maternal educational level, maternal BMI, parenting styles, child ethnic background, child gender, child exact age, days of using childcare and study conditions.

a Results from linear regression model.

b Results from logistic regression model.

## Discussion

We identified three patterns of lifestyle behaviours among 3-year-old children in a population-based sample from the Netherlands: the ‘unhealthy lifestyle’ pattern, the ‘low snacking and low screen time’ pattern, and the ‘active, high fruit and vegetable, high snacking, high screen time’ pattern. Boys, children with lower maternal educational and those being raised with permissive parenting style were more likely to have an ‘unhealthy lifestyle’ pattern, and an ‘active, high fruit and vegetable, high snacking, high screen time’ pattern, compare with girls, children with higher maternal education, and those being raised with authoritative parenting styles. There was no difference in the distribution of BMI or the risk of overweight between the subgroups of children with different lifestyle patterns.

The co-occurrence of unhealthy lifestyle behaviours, including unhealthy dietary intakes, high screen time, and low physical activity level among certain subgroups of children have been noted by various studies among children of different age groups and regions.^9^^,^^11^^,^^32^ These co-occurrence patterns reveal the high correlations between sedentary behaviours (e.g. TV viewing and computer use) and the consumption of sugar-containing drinks and unhealthy snacks. The proposed mechanism for the correlations included the promoting effect of beverage and snack commercials on the consumption of these foods,^33^ and the provision of a context during sedentary activities that promotes passive snacking.^34^ The co-occurrence of unhealthy behaviours is also likely to be a result of parental influence, as this pattern has been shown to exist in the adult population.^35^ Further, parents who allow their child to watch TV may also provide the child sugar-containing drinks and unhealthy snacks.^36^

About half of the children were allocated to the ‘low snacking and low screen time’ group, which is considered as relative healthier group. However, children in this group commonly had insufficient fruit and vegetable consumptions and physical activity level. A similar lifestyle pattern has been observed in a sample of 1773 children aged 3–6 years from eight European countries (the ‘low beverage consumption and low sedentary’ pattern).^37^ Such a pattern might reveal that insufficient fruit and vegetable intakes and low physical activity levels are widespread issues and should be addressed universally in the population. The ‘active, high fruit and vegetable, high snacking, and high screen time’ lifestyle pattern is similar to the ‘sporty media-oriented mixed eaters’ pattern reported in a previous study from Belgian.^32^ Further studies are needed to understand the rationale for the co-occurrence of both healthy and unhealthy intakes, and high screen time and high level of physical activities in this group.

Both the ‘unhealthy lifestyle’ and ‘active, high fruit and vegetable, high snacking and high screen time’ patterns were characterized by high consumptions of unhealthy snacks and sugaring drinks, and high screen time, and the two patterns share similar correlates of parental and child characteristics. The associations between the less healthy lifestyle patterns with lower maternal educational level are accordance with the previous research.^9^ Our results suggest that children with permissive parents are more likely to have lifestyle patterns characterized by high consumptions of unhealthy snacks and sugar-containing beverages, than those with authoritative parents. Our finding is consistent with previous research investigating the associations of parenting style with individual lifestyle behaviours, that an authoritative parenting style is associated with more favourable lifestyles behaviours, while a permissive parenting style is associated with less healthy lifestyles of children.^13^^,^^14^^,^^38^ It is possible that permissive parents are more likely to cater their children’s preferences, e.g. on the consumption of snacks and sugar-containing drinks, and TV/computer watching, rather than place many demands on these behaviours.^13^

Our findings confirm that the co-occurrence of multiple overweight-related lifestyle behaviours can already be observed in children as young as 3 years. Public health practitioners should consider how the lifestyle behaviours co-occur and which background characteristics are associated with the co-occurring patterns of lifestyle behaviours in order to develop better targeted interventions to improve children’s lifestyle behaviours tailored to different groups.

We found no association between the lifestyle patterns and children’s BMI or weight status. Previous findings with regard to the association between lifestyle patterns and children’s weight status are inconsistent^7^^,^^8^^,^^32^^,^^37^^,^^39^: while some studies have found evidence of possible synergistic effect of multiple unhealthy behaviours on childhood overweight,^8^ others found no association,^32^ or even reverse association.^39^ A possible explanation for the lack of association between the lifestyle patterns and children’s BMI or weight status may include the younger age of our study population. The adverse effect of multiple unhealthy lifestyle behaviours on child BMI or weight status may accumulate and manifest at later ages.^40^ This might represent an opportunity for the primary prevention of overweight, as we would be able to prevent the accumulation of unhealthy lifestyle behaviours. In addition, parents may restrict the unhealthy behaviours such as sugar-containing drink consumption and unhealthy snack consumption of the overweight or obese children.^39^ Further, it is a limitation of our study that only BMI was measured to represent the adiposity of children. Since BMI is an indicator for both fat mass and fat free mass, it could be that children in the unhealthy lifestyle group had lower muscle mass and higher fat mass compared with children with relatively healthier lifestyles.^39^

This study was subjected to some limitations. First, information on children’s lifestyle behaviour variables were self-reported by parents through questionnaires. In addition, the questions concerning unhealthy snacks and fruit consumption did not measure serving size. Future studies with more accurate measurement of children’s lifestyle behaviours are warranted to confirm our finding. Second, our study sample is relatively highly educated, containing predominantly Caucasian, compared with those excluded due to loss of follow-up, and therefore caution is needed when generalizing results. Third, variations in the variables included, and the way in which the lifestyle behaviours were dichotomized may hinder the comparison between studies. Fourth, this study used data from an intervention trial. However, we found no association between the intervention groups and the lifestyle patterns (see table 2). In addition, we have replicated the latent class analyses using data from the control group only, and comparable lifestyle patterns were generated (Supplementary table S5). Finally, given the limitation of observational studies, we could not determine the causal relationships but only associations.

## Conclusion

Our findings confirm that co-occurrence of lifestyle behaviours can be observed among children aged 3 years. Lower maternal educational level, permissive parenting style and male gender of the child were associated with having unhealthy lifestyle patterns for the child. We found no association between the lifestyle patterns and children’s BMI or weight status. Our findings underline the importance of designing and implementing interventions that consider the diversity of lifestyle patterns and associated determinants.

## Supplementary data

Supplementary data are available at *EURPUB* online.

## Funding

The BeeBOFT study was funded by a grant from ZonMW, the Netherlands Organization for Health Research and Development (grant number 50-50110-96-491).

*Conflicts of interest*: None declared.

**Key points**

- Clustering of lifestyle behaviour can be observed among children as young as 3 years.
- Lower maternal educational level, permissive parenting style and male gender of the child are associated with having unhealthy lifestyle patterns for the child.
- The diversity of lifestyle patterns and associated determinants should be considered when designing and implementing interventions that aim at improving children’s health behaviours.

## Supplementary Material

ckaa109_supplementary_data

## References

[1] World Health Organization. *Report of the Commission on Ending Childhood Obesity* 2016 Availableat: http://apps.who.int/iris/bitstream/handle/10665/204176/9789241510066_eng.pdf?sequence=1 (1 June 2019, date last accessed).

[2] Valerio G, Licenziati MR, Manco M, et al [Health consequences of obesity in children and adolescents]. Conseguenze dell’obesita sulla salute del bambino e dell’ adolescente. Minerva Pediatr 2014;66:381–414. 25253187

[3] Llewellyn A, Simmonds M, Owen CG, et al Childhood obesity as a predictor of morbidity in adulthood: a systematic review and meta-analysis. Obes Rev 2016;17:56–67. 26440472 10.1111/obr.12316

[4] van Stralen MM, te Velde SJ, van Nassau F, et al; ToyBox-study group. Weight status of European preschool children and associations with family demographics and energy balance-related behaviours: a pooled analysis of six European studies. Obes Rev 2012;13:29–41. 22309063 10.1111/j.1467-789X.2011.00959.x

[5] Pate RR, O’Neill JR, Liese AD, et al Factors associated with development of excessive fatness in children and adolescents: a review of prospective studies. Obes Rev 2013;14:645–58. 23601571 10.1111/obr.12035

[6] te Velde SJ, van Nassau F, Uijtdewilligen L, et al; ToyBox-study group. Energy balance-related behaviours associated with overweight and obesity in preschool children: a systematic review of prospective studies. Obes Rev 2012;13:56–74. 22309065 10.1111/j.1467-789X.2011.00960.x

[7] Leech RM, McNaughton SA, Timperio A The clustering of diet, physical activity and sedentary behavior in children and adolescents: a review. Int J Behav Nutr Phys Act 2014;11:4. 24450617 10.1186/1479-5868-11-4 PMC3904164

[8] Leech RM, McNaughton SA, Timperio A Clustering of diet, physical activity and sedentary behaviour among Australian children: cross-sectional and longitudinal associations with overweight and obesity. Int J Obes 2015;39: 1079–85. 10.1038/ijo.2015.66 25907316

[9] Miguel-Berges ML, Zachari K, Santaliestra-Pasias AM, et al Clustering of energy balance-related behaviours and parental education in European preschool children: the ToyBox study. Br J Nutr 2017;118:1089–96. 29198192 10.1017/S0007114517003129

[10] Santaliestra-Pasias AM, Mouratidou T, Reisch L, et al Clustering of lifestyle behaviours and relation to body composition in European children. The IDEFICS study. Eur J Clin Nutr 2015;69:811–6. 26039315 10.1038/ejcn.2015.76

[11] Huh J, Riggs NR, Spruijt-Metz D, et al Identifying patterns of eating and physical activity in children: a latent class analysis of obesity risk. Obesity (Silver Spring) 2011;19:652–8. 20930718 10.1038/oby.2010.228 PMC5310931

[12] Kremers SP Theory and practice in the study of influences on energy balance-related behaviors. Patient Educ Couns 2010;79:291–8. 20371159 10.1016/j.pec.2010.03.002

[13] Vollmer RL, Mobley AR Parenting styles, feeding styles, and their influence on child obesogenic behaviors and body weight. A review. Appetite 2013;71:232–41. 24001395 10.1016/j.appet.2013.08.015

[14] Sleddens EFC, Gerards SMPL, Thijs C, et al General parenting, childhood overweight and obesity-inducing behaviors: a review. Int J Pediatr Obes 2011;6:e12–27. 21657834 10.3109/17477166.2011.566339

[15] Raat H, Struijk MK, Remmers T, et al Primary prevention of overweight in preschool children, the BeeBOFT study (breastfeeding, breakfast daily, outside playing, few sweet drinks, less TV viewing): design of a cluster randomized controlled trial. BMC Public Health 2013;13:974. 24138805 10.1186/1471-2458-13-974 PMC3840600

[16] van Grieken A, Vlasblom E, Wang L, et al Personalized web-based advice in combination with well-child visits to prevent overweight in young children: cluster randomized controlled trial. J Med Internet Res 2017;19:e268. 28751299 10.2196/jmir.7115 PMC5553002

[17] van Grieken A, Veldhuis L, Renders CM, et al Population-based childhood overweight prevention: outcomes of the ‘Be active, eat right’ study. PLoS One 2013;8:e65376. 23741491 10.1371/journal.pone.0065376 PMC3669240

[18] van der Horst K, Oenema A, van de Looij-Jansen P, et al The ENDORSE study: research into environmental determinants of obesity related behaviors in Rotterdam schoolchildren. BMC Public Health 2008;8:142. 18442370 10.1186/1471-2458-8-142 PMC2413223

[19] Centers for Disease Control and Prevention. *How Much Physical Activity Do Children Need* 2011 Available at: https://www.cdc.gov/physicalactivity/basics/index.htm?CDC_AA_refVal=https%3A%2F%2Fwww.cdc.gov%2Fcancer%2Fdcpc%2Fprevention%2Fpolicies_practices%2Fphysical_activity%2Fguidelines.htm (1 June 2019, date last accessed).

[20] Council on Communications and Media. Media and Young Minds. Pediatrics 2016;138:e20162591. 27940793 10.1542/peds.2016-2591

[21] Tremblay MS, LeBlanc AG, Carson V, et al Canadian sedentary behaviour guidelines for the early years (aged 0–4 years). Appl Physiol Nutr Metab 2012;37:370–80. 22448609 10.1139/h2012-019

[22] Davis MM, Gance-Cleveland B, Hassink S, et al Recommendations for prevention of childhood obesity. Pediatrics 2007;120:S229–53. 18055653 10.1542/peds.2007-2329E

[23] Jeugdgezondheid NC *Guideline: Nutrition and eating behavior (2013, adaptation 2017)* Available at: https://www.ncj.nl/richtlijnen/alle-richtlijnen/richtlijn/?richtlijn=4&rlpag=527 (1 June 2019, date last accessed).

[24] Bulk-Bunschoten AMW, Renders CM, Van Leerdam FJM, HiraSing RA *(Youth health care overweight prevention protocol) Overbruggingsplan voor kinderen met overgewicht* 2005 Available at: https://www.nji.nl/nl/Databank/Databank-Effectieve-Jeugdinterventies/Overbruggingsplan-voor-kinderen-met-overgewicht.html (1 June 2019, date last accessed).

[25] Wake M, Nicholson JM, Hardy P, et al Preschooler obesity and parenting styles of mothers and fathers: Australian national population study. Pediatrics 2007;120:e1520–7. 18055667 10.1542/peds.2006-3707

[26] Bulk-Bunschoten AMW, Renders CM, Van Leerdam FJM, HiraSing RA. *Signaleringsprotocol overgewicht in de jeugdsgezondheidszorg Youth health care overweight-detection-protocol*. 2005. Available at: https://www.ggdghorkennisnet.nl/?file=748&m=1310480599&action=file.download (1 June 2019, date last accessed).

[27] World Health Organization. WHO Child Growth Standards: Length/Height for Age, Weight-for-Age, Weight-for-Length, Weight-for-Height and Body Mass Index-for-Age, Methods and Development. 2006.

[28] Cole TJ Establishing a standard definition for child overweight and obesity worldwide: international survey. BMJ 2000;320:1240. 10797032 10.1136/bmj.320.7244.1240 PMC27365

[29] Cole TJ, Lobstein T Extended international (IOTF) body mass index cut-offs for thinness, overweight and obesity. Pediatr Obes 2012;7:284–94. 22715120 10.1111/j.2047-6310.2012.00064.x

[30] Lanza ST, Collins LM, Lemmon DR, et al PROC LCA: a SAS procedure for latent class analysis. Struct Equ Modeling 2007;14:671–94. 19953201 10.1080/10705510701575602 PMC2785099

[31] Goodman LA Exploratory latent structure analysis using both identifiable and unidentifiable models. Biometrika 1974;61:215–231.

[32] Seghers J, Rutten C Clustering of multiple lifestyle behaviours and its relationship with weight status and cardiorespiratory fitness in a sample of Flemish 11- to 12-year-olds. Public Health Nutr 2010;13:1838–46. 20236562 10.1017/S1368980010000418

[33] Halford JCG, Boyland EJ, Hughes G, et al Beyond-brand effect of television (TV) food advertisements/commercials on caloric intake and food choice of 5–7-year-old children. Appetite 2007;49:263–267. 17258351 10.1016/j.appet.2006.12.003

[34] Coon KA, Goldberg J, Rogers BL, et al Relationships between use of television during meals and children’s food consumption patterns. Pediatrics 2001;107:e7. 11134471 10.1542/peds.107.1.e7

[35] Perez-Rodrigo C, Gianzo-Citores M, Gil A, et al Lifestyle patterns and weight status in Spanish adults: the ANIBES Study. Nutrients 2017;9:606. 10.3390/nu9060606 PMC5490585 28613259

[36] Rodenburg G, Oenema A, Kremers SP, et al Clustering of diet-and activity-related parenting practices: cross-sectional findings of the INPACT study. Int J Behav Nutr Phys Act 2013;10:36. 23531232 10.1186/1479-5868-10-36 PMC3618009

[37] Bel-Serrat S, Mouratidou T, Santaliestra-Pasías AM, et al; on behalf of the IDEFICS consortium Clustering of multiple lifestyle behaviours and its association to cardiovascular risk factors in children: the IDEFICS study. Eur J Clin Nutr 2013;67:848–854. 23632753 10.1038/ejcn.2013.84

[38] Kremers SPJ, Brug J, de Vries H, et al Parenting style and adolescent fruit consumption. Appetite 2003;41:43–50. 12880620 10.1016/s0195-6663(03)00038-2

[39] van der Sluis ME, Lien N, Twisk JW, et al Longitudinal associations of energy balance-related behaviours and cross-sectional associations of clusters and body mass index in Norwegian adolescents. Public Health Nutr 2010;13:1716–21. 20883571 10.1017/S1368980010002272

[40] Gubbels JS, Kremers SPJ, Stafleu A, et al Clustering of dietary intake and sedentary behavior in 2-year-old children. J Pediatr 2009;155:194–8. 19394036 10.1016/j.jpeds.2009.02.027

