# Supplementary material for: Identifying patterns of lifestyle behaviours among children of 3 years old
Source: Eur J Public Health. 2020 Jul 12;30(6):1115–21. doi: 10.1093/eurpub/ckaa109 (PMC7733046; doi:10.1093/eurpub/ckaa109)
Supplement: ckaa109_supplementary_data [file ckaa109_supplementary_data.docx]

**Table S1.** Non-response analyses.

|  | Population for analyses (n=2090) | Population Excluded (n=913) |  |
| --- | --- | --- | --- |
| Variables | %/ mean(SD) | %/ mean(SD) | *p-value* |
| Maternal educational level |  |  | <0.001 |
| ≤4 years secondary school | 10.14 | 22.27 |  |
| >4 years secondary school or middle level vocational training | 35.29 | 37.91 |  |
| University or above | 54.57 | 39.82 |  |
| Maternal weight status baseline |  |  | 0.15 |
| Normal | 12.11 | 14.78 |  |
| Overweight | 31.94 | 30.51 |  |
| Obesity | 55.95 | 54.71 |  |
| Child ethnic background, Dutch^1^ | 84.11 | 80.37 | 0.01 |
| Child gender, male | 51.48 | 49.49 | 0.32 |
| Intervention group |  |  | 0.003 |
| Control group | 34.89 | 39.96 |  |
| ‘BBOFT+’ | 29.68 | 30.74 |  |
| ‘E-health4Uth’ | 35.42 | 29.31 |  |
| Parenting style |  |  |  |
| Authoritative | 40.34 | 31.43 | 0.10 |
| Permissive | 14.7 | 15.24 |  |
| Authoritarian | 25.74 | 24.76 |  |
| Permissive | 19.22 | 28.57 |  |

^1^A child’s ethnic background was defined according to the ethnic backgrounds of his/her parents. A parent was classified as non-Dutch if one of his/her own parents was born outside the Netherlands. If one or both of the child’s parents were classified as non-Dutch, that child’s ethnic background was non-Dutch

**Table S2.** The questionnaire for child lifestyle behaviors at child age 36 months.

|  | Answering categories and value assignment |
| --- | --- |
| **Sugar-containing beverages consumption** |  |
| A1. How many cups or packages does your child drink on average on a weekday of the sugar-containing beverages (examples of sugar-containing beverages were provided: soft drinks such as cola, 7-ups … Ice-tea, fruit cordials or syrup; fruit juice, sweetened fruit juice beverages; sport and energy drinks with sugar; sweetened dairy drinks; tea or water with sugar) | No or lower than 1=0, 1, 2, …, 9 or more=9; |
| A2. How many cups or packages does your child drink on average on a weekend day of the Sugar-containing beverages? | No or lower than 1=0, 1, 2, …, 9 or more=9; |
| Calculation: Child sugar-containing drinks consumption per day=(A1*5+A2*2)/7 | |
| **Unhealthy snacks consumption** |  |
| B1 How many servings of the following snacks does your child eat on average one day? Candy (cake, ice cream, chocolate, candies, mars) | 0 or less than 1=0, 1, 2, …, 9 or more=9; |
| B2 How many servings of the following snacks does your child eat on average one day? Snacks (chips, nuts, fries, pizza, hamburger) | 0 or less than 1=0,1, 2, …, 9 or more=9; |
| Calculation: Child unhealthy snack consumption per day=B1+B2 | |
| **Fruit consumption** |  |
| How many servings of fruit does your child eat on average per day? (examples of fruits provided. apple, orange, pear, kiwi, banana, mandarin, strawberry) | No intake, less than ½ serving, ½ serving, 1 serving, 2 serving, 3 serving, 4 serving, 5 serving or more; |
| **Vegetable consumption** |  |
| How many serving spoons of vegetable does your child eat on average per day? (examples of vegetables provided: Green beans, Carrots, Cauliflower, Sweet pepper, Broccoli, Red cabbage, Sprouts, Tomato, Lettuce, Kale, Spinach, Leek, Cucumber) | No intake, less than ½ serving, ½ serving, 1 serving, 2 serving, 3 serving, 4 serving, 5 serving or more; |
| **Physical activity** |  |
| How many days a week does your child engage in activities that he/she can move actively? (for example: Swimming, toddler gym, movement to music, walking to the store) Ordinary playing at home we do not count here. | Less than one day, 1 day, 2 days, 3 days, …, 6 days, everyday |
| How many hours per day on average does your child engage in activities that he/she can move actively? | Not applicable  less than 30 minutes per day  30 minutes to 1 hour per day  1 to 2 hours per day  2 to 3 hours per day  More than 3 hours per day |
| **Screen time** |  |
| C1. Does your child watch TV/video/DVD? | NO=0, Yes=1; |
| C2. On how many days does your child watch TV/video/DVD during the weekdays (number of days)? | Not applicable=0, less than one day=0.5, 1 day=1, 2 days=2, 3 days=3, 4 days=4, 5 days=5; |
| C3. How long per day does your child watch TV/video/DVD on average during the weekdays (hours)? | Not applicable=0,  less than 30 minutes per day =0.25,  30 minutes to 1 hour per day=0.75,  1 to 2 hours per day =1.5,  2 to 3 hours per day =2.5,  3 to 4 hours per day =3.5,  4 to 5 hours per day =4.5,  5 to 6 hours per day =5.5,  more than 6 hours=6.5; |
| C4. On how many days does your child watch TV/video/DVD during the weekends (number of days)? | Not applicable=0, less than one day =0.5, 1 day=1, 2 days=2; |
| C5. How long per day does your child watch TV /video/DVD on average on weekends (hours)? | Not applicable=0,  less than 30 minutes per day =0.25,  30 minutes to 1 hour per day=0.75,  1 to 2 hours per day =1.5,  2 to 3 hours per day =2.5,  3 to 4 hours per day =3.5,  4 to 5 hours per day =4.5,  5 to 6 hours per day =5.5,  more than 6 hours=6.5; |
| C6. Does your child play computer games? | NO=0, Yes=1; |
| C7. On how many days does your child spend time on playing computer games during the weekdays (number of days)?? | Not applicable=0, less than one day=0.5, 1 day=1, 2 days=2, 3 days=3, 4 days=4, 5 days=5; |
| C8. How long per day does your child spend on playing computer games on average during the weekday (hours)? | Not applicable=0,  less than 30 minutes per day =0.25,  30 minutes to 1 hour per day=0.75,  1 to 2 hours per day =1.5,  2 to 3 hours per day =2.5,  3 to 4 hours per day =3.5,  4 to 5 hours per day =4.5,  5 to 6 hours per day =5.5,  more than 6 hours=6.5; |
| C9. On how many days does your child spend time on playing computer games during the weekends (number of days)? | Not applicable=0, less than one day =0.5, 1 day=1, 2 days=2; |
| C10. How long per day does your child play computer games on average during the weekends (hours)? | Not applicable=0,  less than 30 minutes per day =0.25,  30 minutes to 1 hour per day=0.75,  1 to 2 hours per day =1.5,  2 to 3 hours per day =2.5,  3 to 4 hours per day =3.5,  4 to 5 hours per day =4.5,  5 to 6 hours per day =5.5,  more than 6 hours=6.5 ; |
| Calculation: Child average screen time per day =(C2*C3+ C4*C5)/7+ (C7*C8+C9*C10)/7 | |

Note: Parents were asked to keep in mind the average condition of the child in the past four weeks.

**Table S3.** The questionnaire for measuring parenting style in the present study

| **Parental Warmth** |
| --- |
| How often do you express affection by hugging, kissing, and holding this child? |
| How often do you hug or hold this child for no particular reason? |
| How often do you tell this child how happy he/she makes you? |
| How often do you have warm, close times together with this child? |
| How often do you enjoy doing things with this child? |
| How often do you feel close to this child both when he/she is happy and when he/she is upset? |
| **Parental Control** |
| When you give this child an instruction or make a re- quest to do something, how often do you make sure that he/she does it? |
| If you tell this child he/she will get punished if he/she doesn’t stop doing something, but he/she keeps doing it, how often will you punish him/her? |
| How often does this child get away with things that you feel should have been punished? (R) |
| How often is this child able to get out of punishment when he/she really sets his/her mind to it? (R) |
| When you discipline this child, how often does he/she ignore the punishment? (R) |

Responses were on 5-point Likert scales and ranged from 1 (“never/ almost never”) to 5 (“all the time”). The items were summed to calculate the score for each dimension.

**Table S4.** Model fit statistics of the latent class models according to the number of classes.

| Number of latent classes | Loglikelihood | Akaike’s Information Criterion | Bayesian Information Criterion |
| --- | --- | --- | --- |
| 1 | -8333.36 | 297.04 | 311.85 |
| 2 | -8235.86 | 116.04 | 148.12 |
| 3 | -8215.95 | 90.21 | 139.57 |
| 4 | -8209.36 | 91.03 | 157.66 |
| 5 | -8206.05 | 98.42 | 182.33 |
| 6 | -8202.29 | 104.89 | 206.08 |

**Table S5.** The probabilities of reporting each unfavorable lifestyle behaviors in the total sample and conditional on the latent classes: results from the control group only.

| Unfavorable lifestyle behaviors | Overall  Sample  (N=730) | Class 1  “unhealthy lifestyle”  (27%) | Class 2  “low snacking and low sedentary”  (48%) | Class 3  “active, high vegetable, high snacking and high screen time”  (24%) | *p-value*^1^ |
| --- | --- | --- | --- | --- | --- |
| Sugar-containing drinks> 2 cups per day | 0.41 | 0.63 | 0.10 | 0.78 | <0.001 |
| Unhealthy snacks >1 serving per day | 0.41 | 0.60 | 0.15 | 0.72 | <0.001 |
| Fruit intake ≤1 servings per day | 0.57 | 0.76 | 0.46 | 0.58 | <0.001 |
| Vegetable intake ≤1 serving spoons per day | 0.66 | 1.00 | 0.62 | 0.37 | <0.001 |
| Screen time >1 hours per day | 0.47 | 0.71 | 0.18 | 0.80 | <0.001 |
| Physical activity <1 hour per day | 0.63 | 1.00 | 0.62 | 0.26 | <0.001 |

^1^ The difference between groups were compared using Chi-square test.
